# Micro- and Nanoparticulate Hydroxyapatite Powders as Fillers in Polyacrylate Bone Cement—A Comparative Study

**DOI:** 10.3390/ma13122736

**Published:** 2020-06-17

**Authors:** Anna Sobczyk-Guzenda, Paulina Boniecka, Anna Laska-Lesniewicz, Marcin Makowka, Hieronim Szymanowski

**Affiliations:** Institute of Materials Science and Engineering, Lodz University of Technology, Stefanowskiego 1/15 Str., 90-924 Lodz, Poland; 233435@edu.p.lodz.pl (P.B.); anna.laska-lesniewicz@dokt.p.lodz.pl (A.L.-L.); marcin.makowka@p.lodz.pl (M.M.); hieronim.szymanowski@p.lodz.pl (H.S.)

**Keywords:** bone cement, hydroxyapatite, nanoscale filler, microscale filler, acrylate copolymer, morphology, cross-linking time

## Abstract

Acrylate polymer-based bone cements constitute the most popular bonding agents used in regenerative surgery. Due to their inferior biocompatibility, however, these materials are often enriched with ceramic additives including hydroxyapatite (HAp). The aim of this paper was to perform a comparative study of the acrylate cements filled with different content (3–21%) of nano- and microscale hydroxyapatite. The work concerns a comparison of times and temperatures of the cross-linking reaction, as well as morphology, glass transition temperature, and principal mechanical properties of the resulting composites. Before being used as a filler, both HAp forms were subjected to an in-depth characterization of their morphology, specific surface area, pore size distribution, and wettability as well as chemical composition and structure. For that purpose, such analytical techniques as scanning electron microscopy, Fourier-transform infrared spectroscopy, X-ray diffraction, tensiometry, Brunauer–Emmett–Teller surface area analysis, differential scanning calorimetry, Shore D hardness test, and Charpy impact test were used. The results indicated a drop of cross-linking temperature and an extension of setting time with the addition of µHAp. The µHAp-filled acrylate composites were characterized by a globular surface morphology, higher glass transition temperature, and lower hardness and impact strength compared to nHAp-filled materials. This relationship was evident at higher nHAp concentrations.

## 1. Introduction

A bone has an ability to self-repair. Nevertheless, in certain cases, this mechanism is insufficient. Due to a high demand for tissue replacement and strengthening, either synthetically derived or naturally grown materials have been extensively studied for more than a decade [1]. Currently, the fabrication of synthetic materials that possess the same properties as natural bone is under a constant development. The new solutions seek to combine the bone formation and material resorption, taking into consideration individual factors such as patient age, gender, or social habits [2]. A group of such materials is comprised of bone cements. Due to a high demand for bone cements in joint replacement and spinal surgery procedures, there exist many variations of these materials regarding their viscosity, contrast agent, and the presence of other additives. This allows one to choose the most reliable product for a given application [3]. Poly(methyl methacrylate) (PMMA) is the most frequently used bone cement in implants. By getting into the crevices of a bone, it assures its mechanical fixing [4]. In a cement arthroplasty, its role is to transfer body weight and service loads from the prosthesis to the bone [5]. From the economic point of view, in comparison with other substance-based bone cements, it is rather inexpensive [6]. However, it has certain drawbacks. One of them is a poor biocompatibility in contact with human tissues. Therefore, hydroxyapatite is added to improve biotolerance and to make the material similar to that of human bones [7]. Calcium phosphate (CaP) ceramics are characterized by significantly high biocompatibility, bioactivity, and biodegradation, making it one of the major classes of biomaterials serving as a bone scaffold. Besides, it has an open, porous structure that promotes the bone to grow into free spaces. However, the formation of pores has shortcomings such as diminishing mechanical properties. Due to their similarity to natural bone, tricalcium phosphate (TCP) and hydroxyapatite (Ca_10_(PO_4_)_6_(OH)_2_) are the most frequently used CaP materials. Hydroxyapatite (HAp) can easily take the form of a powder [8,9]. Advantageous physical and chemical properties of HAp contributed to its rapid conquest of the medical field. Apart from its nontoxicity, it exhibits an outstanding biocompatibility with human tissue. In addition, it is biodegradable and viscoelastic and, when added to a composite, it assures better cell attachment to the scaffold. All the features mentioned above are closely related to the material crystallinity as well as to the size and surface morphology of its crystals and contribute to the changes of their mechanical properties [10].

A formation of HAp/polyacrylate (PAc) composite is supposed to result in a set of properties derived from both materials. Separately, PAc and HAp possess unwanted features, namely high brittleness and low strength [11]. Research has shown that the composite exhibits improvement in cell proliferation and biological stability. Moreover, the amelioration in osteoconductivity and bone regeneration ability is also observed [12]. HAp/PMMA cements possess compressive strength that is high enough to ensure an appropriate growth of a tissue and a composite implantation. However, a higher concentration of HAp and larger porosity lead to the decrease of mechanical strength. It is also known that the size of HAp particles has an impact on composite characteristics [7,13].

The introduction of nanoparticles, i.e., particles of the size between 1 and 100 nm, into composite materials’ technology brought a breakthrough due to their superior mechanical and biological properties. They are complementary to microparticles, since they fill up spaces between them, thus contributing to their reinforcement [14]. Nanohydroxyapatite gained interest in many fields of medical science, especially in dentistry. Being used as a toothpaste additive, it exhibits an ability to bind proteins and bacteria, owing to larger surface area [15]. Moreover, a nanoform of HAp (nHAp), just as it is in the case of bigger particles, is characterized by good biocompatibility and bone integration [16]. According to the literature, smaller particle size of HAp results in stiffer composites. This is a reason why an addition of nHAp enhances static and dynamic mechanical characteristics [17]. Since bioceramics/biopolymer composite is considered to be the best material to create bone grafts, the combination of nHAp with natural compounds is being widely studied [18]. Also, research concerning different processing forms of HAp such as microspheres, nanofibers, or three-dimensional scaffold particles is being conducted. Nevertheless, there are barely any publications in the literature that report an effect of the particle size of inorganic filler in the form of HAp on mechanical, biological, and physico-chemical properties of PMMA-based composites.

The aim of the study was to investigate the impact of particle size, chemical structure, specific surface area, and degree of crystallinity of hydroxyapatite filler introduced into the acrylate copolymer (PAc) matrix on its morphology, physico-chemical, and mechanical properties. The work consists of two parts. The first part is devoted to the investigation of HAp nano- and micropowders, while the second one will characterize the nHAp/PAc and µHAp/PAc composite materials obtained.

## 2. Materials and Methods

### 2.1. Materials

Nanohydroxyapatite

Sigma—Aldrich commercial hydroxyapatite of nanopowder size, relative density of 3140 g/cm^3^, and XRF (X-ray fluorescence spectroscopy) purity ≥97% was used in this work.

Microhydroxyapatite

Powder was obtained by a wet precipitation method elaborated by Afshar and coworkers [19]. The method is most frequently used on an industrial and laboratory scale due to its capability of obtaining the powder of appropriate morphology, crystalline structure, and molar ratio close to stoichiometric HAp [20]. An additional advantage is a release of water as a by-product. The reaction takes place according to the following equation.

$$10\mathrm{Ca}{\left(\mathrm{OH}\right)}_{2}+6H_{3}{\mathrm{PO}}_{4}\to Ca_{10}{\left({\mathrm{PO}}_{4}\right)}_{6}{\left(\mathrm{OH}\right)}_{2}+18\;H_{2}O$$

The reaction was conducted at the temperature of 40 °C for 1 h. Throughout that entire period of time the pH of the reaction mixture was maintained at 12. After a complete dissolution of the calcium hydroxide, H_3_PO_4_ phosphoric acid was added dropwise at the rate of 1–2 drops per second, while maintaining the temperature at 40 °C and pH above 9, (by means of dosing ammonia water solution). The obtained mixture was left for 24 h for the sedimentation of hydroxyapatite. The resulting suspension was rinsed with distilled water in order to assure a proper purity of HAp.

The precipitate was filtered and subsequently dried at the temperature of 25 °C. Resulting hydroxyapatite was initially grinded in a ceramic mortar and then the grinding procedure was repeated using a Retsch PM-100 planetary-ball mill, which allowed for a high degree of fragmentation of the raw material. Shredded HAp was placed in a sieve laboratory shaker to obtain the particle size fraction of 0.05–0.075 mm. PMMA (methyl methacrylate) and PMA (methyl acrylate) copolymer powder was used as a polymer base. In the first part of the work, we introduced the acronym PAc (polyacrylate) for polymer powder (under the trade name Duracyl Plus from SpofaDental company) serving as a polymeric base.

### 2.2. Methods

#### 2.2.1. HAp Powders

##### Morphology and Chemical Composition Measurements

Surface morphology of the powders was determined by means of a scanning electron microscopy (SEM) with chemical composition being established with the help of the X-ray energy dispersive spectroscopy method (EDS).

The morphology of HAp particles was determined with a use of JEOL JSM-6610LV scanning electron microscope (JEOL Ltd., Tokyo, Japan) working in low vacuum mode with Oxfords Instruments X-MAX 80 EDS module attached (Oxford Instruments NanoAnalysis & Asylum Research, High Wycombe, UK). For morphology investigations, the following parameters were used: Accelerating voltage of 20 kV, pressure of 50 Pa, and beam current (spot size) of a medium value, set for the best sharpness of the image. Elemental composition was recorded using the same parameters i.e., the resolution of 1024 (number of pixels in the X dimension over which the beam scans) and pixel dwell time of 100 µs. Samples of pure HAp powders used as additions in a composite preparation were examined in terms of their size distribution. For that purpose, detailed SEM images of powders were taken using the following parameters: High vacuum working mode and accelerating voltage of 20 kV. For the image processing, ImageJ 1.50i software was used with the results being statistically analyzed with the help of OriginPro 9.0.0 software (Originlab Corporation, Wellesley Hills, MA, USA).

##### Chemical Structure Analysis Using Fourier-Transform Infrared Spectroscopy (FTIR)

Chemical structure analysis was carried out with the help of a Nicolet INs50 FTIR spectrometer (Thermo Fisher Scientific, Waltham, MA, USA) using Seguell DRIFT reflection/scattering attachment with a variable angle of incidence of the radiation beam. Samples of HAp powders were mixed with potassium bromide (KBr) in a 1:100 proportion and placed in a holder dedicated to the reflection attachment. Measurements were carried out in an absorbance mode within the wavenumber range of 4000–450 cm^−1^ with a resolution of 4 cm^−1^ at the IR beam angle of 90 deg. The number of scans was equal to 128.

##### Wetting Angle Measurements

The measurements of the surface free energy (SFE) and of its polar and dispersive components were performed with the help of a model K100 MKII tensiometer (KRÜSS GmbH, Hamburg Germany). The values of contact angle were determined using polar (methanol) and nonpolar (n-heptane) liquids. As the final step of an assessment, the magnitudes of surface free energy (SFE) and its components were calculated by applying the Owens–Wendt–Rabel–Kaeble formalism.

##### Brunauer–Emmett–Teller (BET) Specific Surface Area Analysis

A determination of powder specific surface area was carried out by the low-temperature nitrogen adsorption measurements using Sorptomatic 1900 device (Carlo Erba Instruments). Prior to the measurement, the samples were degassed for 4 h at 200 degrees Celsius under pressure of 10^−4^ Pa. The test was conducted at liquid nitrogen temperature (77K) within the relative pressure (*P*/*P*_0_) range of 0.04 to 1. The specific surface areas of nHAp and µHAp were determined using the Brunauer–Emmett–Teller equation.

A linear form of the equation is as follows:

(1) $$\frac{1}{W\;\left(\;P/P_{0})-1\right)}=\frac{1}{C\;W_{m\;}}+\frac{C-1}{C\;W_{m}}\left(\frac{P}{P_{0}}\right)$$

where *P/P*_0_ denotes relative pressure, *W* denotes mass of the adsorbate, *C* denotes a constant dependent on a difference between the adsorption heat for the first layer and the adsorbate condensation heat, and *W_m_* denotes mass of adsorbate adsorbed in a form of a monolayer.

A determination of *W_m_* from the above equation allows one to calculate Brunauer–Emmett–Teller specific surface area (*S_BET_*) based on the equation:

(2) $$S_{BET}=\frac{\;W_{m\;}N\;A_{CS}}{M}$$

where *S_BET_* denotes specific surface area, *N* denotes Avogadro number, and *A_cs_* denotes surface area occupied by one molecule of adsorbate in a monolayer, a so-called sitting area.

Nitrogen was used in the study with its *A_CS_* set at 0.162 nm.

A distribution function of pore volume in prepared samples was calculated using the Barrett–Joyner–Halenda (BJH) relation. The measurements were performed for isotherm adsorption range for pores with a diameter higher than 0.7 nm.

#### 2.2.2. HAp/PAc Composites

##### Preparation of Composites

The bone cement liquid phase was made of 98% of hydroquinone containing MMA (methyl methacrylate) and 2% of dimethyl propiothetin (DMPT), whereas the powder phase contained 1% of di-benzoyl peroxide (BPO) and PMMA/PMA (Duracyl Plus) copolymer in percentage related to the amount of introduced HAp. In order to observe possible changes in the structure of the cement, 3, 6, 12, 18, and 21 weight percent of nHAp and µHAp was added to the powder phase. Powder components were weighed separately with the use of analytical balance of 0.0001 g precision and further placed in a beaker. They were blended in a plenary ball grinder (Retsch, PM 100) using milling balls (diameter of 1 mm, material: ZrO_2_) for 15 min with 100 rotations per minute to assure appropriate degree of distribution/mixing of individual components. Liquid constituents were measured with pipette and combined together in another beaker. Subsequently, the liquid was poured into the powder and stirred manually for 45 to 60 s. As soon as it took the form of a paste, it was transferred to an appropriate mold and left for a complete hardening of the material.

##### Determination of Cross-Linking Time and Cross-Linking Temperature

The rate of the cross-linking process is particularly important during surgery since it defines the time that surgeons have to optimally place an implant. A precise application of bone cement makes the prosthesis more stable and prolongs its lifespan [21]. Setting time was determined while preparing the samples for Shore D test. It was measured since the end of blending until complete hardening of the material. In a properly prepared pellet with a diameter of 1.5 cm, made of the tested material, the temperature, during its exothermic cross-linking, was measured with the help of a thermocoupler. The measurement was carried out at the temperature of 25 °C.

##### Surface Morphology and Analysis of the Distribution of Individual Components Constituting the Composites via SEM and EDS Techniques

The SEM microscope (JEOL JSM-6610LV) was operated under low vacuum (50 Pa) with a typical current of 10 kV and beam current (spot size) of medium value, set for the best focus of an image. Micrographs of the samples were recorded within a magnification range of 200–2500×. For the analysis of component distribution, SEM microscope coupled with EDS X-MAX 80 mm^2^ was used. Besides the quantitative content, a map of elemental distribution was also performed for each specimen.

##### Thermal Characterization by Differential Scanning Calorimetry (DSC) Analysis

Differential scanning calorimetry (DSC) measurements were performed with the help of DSC 204F1 Phoenix instrument (NEZTSCH, Selb, Germany), with nitrogen as a purge gas at a flow rate of 250 mL/min, heating and cooling rates of 10K/min, sample masses of about 10–13 mg, and aluminum sample pans. The specimens were heated up from 25 °C to 300 °C and held at this temperature for 5 min, then cooled down to room temperature and held for another 5 min. Each sample was then reheated to 300 °C with 10 K/min. DSC plots were analyzed using NEZTSCH Proteus Thermal Analysis software. The glass transition temperature (Tg) was determined from the second heating run in the DSC plot and the value was calculated by the onset of the heat flow change.

##### Mechanical Properties: Shore D Hardness and Charpy Impact Tests

Shore D test is a standardized test based on the measurement of the penetration depth of an indenter in order to assess the resistance of a material. The measurements were performed manually according to PN-ISO 868 standard (Polish Norms-International Organization for Standardization), using MC-DX/D Durometer from Max: Control company, Raciborz, Poland. The durometer indenter foot penetrated each type of prepared composites 10 times (5 times one sample and 5 times another of the same kind). The results were averaged and standard deviations were determined.

A Charpy impact test machine (Cometech Testing machines Co., Ltd, Taichung City, Taiwan) is composed of a pendulum, which is initially raised and locked. After releasing a striker, the pendulum, moving under the influence of a gravitational force, hits a specimen placed in a holder and breaks it. At the moment of breakage under the impact load, the energy absorbed by the sample is measured and its magnitude is recorded. Three samples of each type were tested to obtain an average value of the impact resistance. Measurements were performed on an impact hammer (Dynstat) with the accuracy of 0.005 Joule.

## 3. Results and Discussion

The introduction of hydroxyapatite phase directly into a polymer one allows obtaining bioactive implants that can fulfill specific biomechanical functions. Depending on the form in which the inorganic filler is introduced, it will interact with the polymer matrix to a varying degree. It should be affected by the following factors: Chemical structure of the filler, particle size, crystallinity, surface development (specific surface area), pore size, and surface free energy. For this reason, before discussing the impact of an inorganic filler particle size on the morphology, as well as physico-chemical and mechanical properties of the HAp/PAc composite, it is essential to characterize the powders before blending them with the polymer matrix.

### 3.1. Characterization of HAp Powders

**Figure 1:**
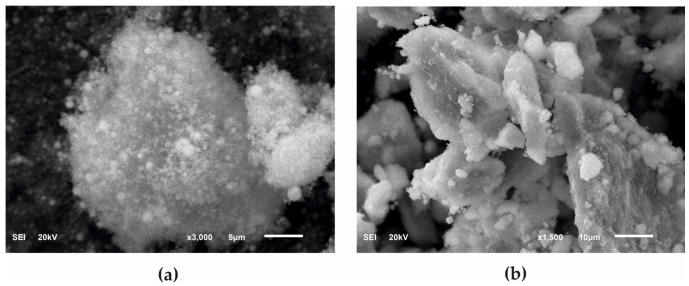
SEM images of HAp powders: (**a**) Nanosize particles and (**b**) microsize particles.

Figure 1 presents a comparison between the surface morphology of commercial powder with nanoscale HAp particles and a powder obtained with the wet method and characterized by micrometer-scale particles (μHAp). For a more accurate assessment of the powder surface condition, the images of powders recorded at two different magnifications, namely 3000× (for nHAp powder) and 1500× (for μHAp powder), are shown. The nHAp sample consisted of either spherical or semicircular particles and it tended to form larger, unstable agglomerates. Their size ranged from 0.02 to 2.33 µm and the average of these measurements equaled 0.24 µm. In contrast, µHAp particles mainly acquired a form of flakes of a very broad size distribution with some smaller particles having the shape of round balls. An image processing procedure demonstrated their size to range between 2.5 µm and 85 µm. However, it should be emphasized that the majority of the particles were small, with their average size amounting to 8.8 µm. This result was over 40 times higher than that of nHAp. In addition, the results of chemical composition measurements performed with the help of EDS microanalysis demonstrated that none of the tested materials contained foreign contaminants. The Ca/P atomic ratio deviated from the stoichiometric one, characteristic for hydroxyapatite. For both powder types it was lower than 1.67—for HAp with nanoscale particles it amounted to 1.62 and for the powder of a micrometer size it was equal to 1.58. This implied a deficiency of calcium with regard to phosphate groups, whereas the preservation of a neutral electrical charge of the entire crystal lattice was possible because of ionic substitution. As a result, an increase in the amount of HPO_4_^2−^ ions was observed in the so-called B-sites of hydroxyapatite crystals, i.e., those originally occupied by PO_4_^3−^ ions.

**Figure 2:**
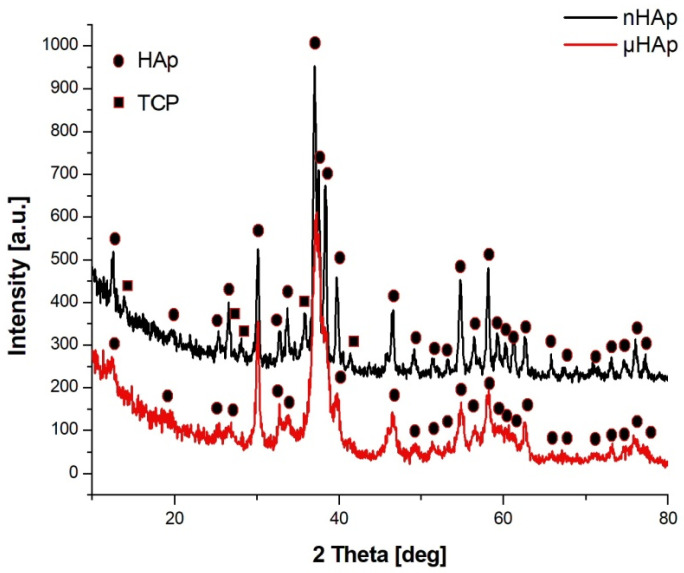
X-ray diffractograms of the nHAp and µHAp powders tested.

Figure 2 shows the X-ray diffractograms of the two tested powders, with their waveforms similar in both cases. In the case of nHAp powder, the dominant phase was hexagonal HAp, but in that powder’s structure there was also a small amount of triphosphate (TCP) of an orthorhombic order. There were no reflections from TCP in the μHAp powder, with only crystallographic systems characteristic for HAp recorded. According to the International Centre for Diffraction Data (ICDD) card with the reference code of 01-073-6113, the hydroxyapatite unit cell had the following dimensions: a = 9.4320 Å, b = 9.4320 Å, c = 6.8810 Å, while the same dimensions for triphosphate were as follows: a = 15.2200 Å, b = 20.7100 Å, c = 9.1090 Å (a card with the reference code 00-009-0348). A small amount of the amorphous phase was also found in both powders. The fact that hydroxyapatite crystallized in the hexagonal system indicated that its structure was non-stoichiometric, thus becoming similar (in terms of its physico-chemical and biological properties) to natural hydroxyapatite.

**Figure 3:**
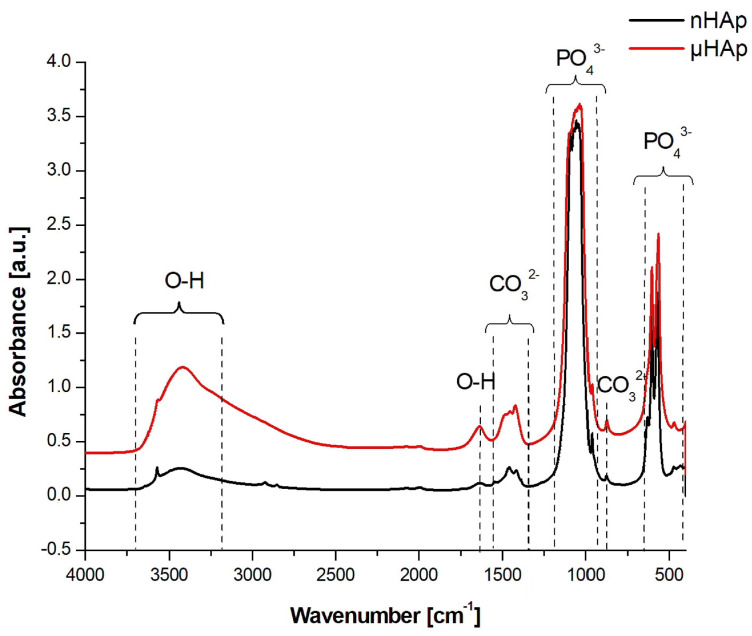
FTIR spectra in the range of 4000–450 cm^−1^ recorded for HAp powders with nano- and microscale particles.

Structural studies of the powders were also carried out using FTIR spectroscopy, with the respective spectra presented in Figure 3. In the course of these spectra, absorption bands corresponding to the PO_4_^3−^ phosphate group could be identified, derived from the vibrations of P-O bonds, a symmetric stretching at 476 cm^−1^, asymmetric bending at 570 cm^−1^ and 603 cm^−1^, symmetric stretching at 965 cm^−1^, and asymmetric stretching at 1020 cm^−1^ and 1085 cm^−1^ [22].

In the spectrum of the nHAp powder a maximum at 1385 cm^−1^ was identified, originating from N-O bond vibrations of the NO^3−^ group, which did not appear in the spectrum of the microscopic particle powder. A wide band having its maximum at a wave number of 1636 cm^−1^ and 3200 cm^−1^ was associated with the presence of water adsorbed on the surface. Both bands were much stronger in the spectrum of the μHAp powder. The wide band between 3400 cm^−1^ and 3200 cm^−1^ originated from OH^−^ groups incorporated into the HAp structure and it was characterized by much higher intensity for self-synthesized hydroxyapatite. A presence of two maxima at 630 and 3573 cm^−1^, characteristic for the apatite environment, was also associated with the OH^−^ groups [22,23].

In the spectrum presented in Figure 3, within the range of 1550–1400 cm^−1^ as well as at the wavenumber of 875 cm^−1^, the presence of carbonate groups was recognized and identified with the respective stretching and bending vibrations of carbon-oxygen bonds, despite the fact that none of the substrates had carbonate or carboxyl groups in their structure [24,25]. These types of bonds are formed naturally in synthetic HAp through the reaction of atmospheric carbon dioxide with the solution used for the HAp synthesis. Carbonates are also found in natural hydroxyapatite in human bones. Their amount ranges from 2 to 8% by weight/volume (w/v) and it is dependent on the type of bone, pathological calcification, or age. As the number of carbonate groups increases, the crystallinity of HAp decreases, thus improving its solubility and leading to the reformation of a bone turnover. The carbonate anion can substitute two anionic HAp structures, thus replacing PO_4_^3−^ groups (B-type apatite) and OH^−^ groups located along the crystallographic c-axis (A-type apatite). A presence of both types of apatite was recorded. However, while in natural apatites a content of A-type carbonate fraction is very low, synthetically produced apatites contain a much higher contribution, reaching up to 6% of this component. The presence of both A and B carbonate fractions can be determined in HAp using the FTIR spectroscopy [24,25]. Baxter et al. [26] identified fraction B by assigning asymmetric stretching vibrations of these bonds to the IR absorption bands at the wavenumbers of 1423 and 1456 cm^−1^. A weak peak originating from the most stable B carbonate fraction may also be recorded at 1410 cm^−1^. When studying HAp that contained only fraction A carbonate, Elliott and Bonel [27,28] confirmed its presence by assigning the maxima at the wave numbers of 1530 and 1465 cm^−1^. Elliot et al. [28], in turn, reported that both A and B fractions might be present in the material wherein a peak at 1543 cm^−1^ and a strong maximum at 1449 cm^−1^ were recorded. When analyzing the spectra shown in Figure 3, one can conclude that μHAp contained type B carbonate groups substituted in the HAp structure by phosphate groups as evidenced by a presence of the maxima found at the following wave numbers: 873, 1421, and 1480 cm^−1^. Furthermore, an additional band at 1480 cm^−1^ was known to be present, which was responsible for the existence of labile surface carbonate [29]. In turn, a presence of fraction B observed in the nHAp powder was also confirmed by the appearance of absorption maxima at the wave numbers of 1414 and 1454 cm^−1^. At the same time, the peak at 1414 cm^−1^ was associated with the most stable form of B carbonate. In addition, peaks at 1448 and 1546 cm^−1^ were also recorded in the spectrum of this material, which suggests that carbonate anions may substitute HAp hydroxyl groups, a feature being associated with the presence of form A. This implies that, in the case of nHAp powder, we dealt with a mixed AB fraction. To summarize, a presence of carbonate groups should not be understood as a contamination since it has been proven that they enhance the bioactivity of hydroxyapatite [30].

When designing polymer-based composite materials, it is extremely important to know the surface wettability of the phases that will be blended together. The degree of dispersion of the inorganic filler in the polymer matrix is dependent upon it, and this, in turn, will translate into the composite mechanical, physico-chemical, and biological properties. For that purpose, the wettability measurements of both powders as potential fillers for the production of HAp/PAc composites were performed. Based on the results of this measurement performed using two liquids, one polar (methanol) and another nonpolar (heptane), the values of surface free energy (SFE) as well as its polar and dispersion components were calculated. The results are shown below, in Table 1.

The results presented in Table 1 indicate that, despite substantial differences in a grain size and identified variations of the chemical structure and phase composition of the tested fillers, no significant effect of these parameters on the SFE value was observed. The resulting value for the powder in the nanoscale was 23.2 mN/m, while that of the microscale material was greater by only 0.3 mN/m. The dispersion component of SFE was similar in both cases, with a minor difference observed for the polar component. A slightly higher value was obtained for the powder with particles on a microscale, which was consistent with the chemical structure results showing a larger content of polar groups in this material.

**Figure 4:**
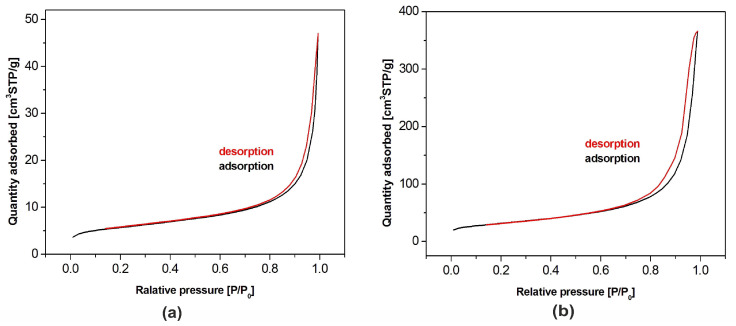
Nitrogen adsorption-desorption isotherms for HAp powders of: (**a**) Nanosize particles and (**b**) microsize particles.

BET adsorption isotherms of both hydroxyapatite powders are shown in Figure 4. According to the International Union of Pure and Applied Chemistry (IUPAC) classification, they belong to the type IV. This means that the tested samples had, apart from recorded microporosity (pore size lower than 2 nm), also well-developed mesoporosity (pore size in the range of 2–50 nm). The isotherms presented in the figure allowed one to calculate the pore size distribution confirming their classification as the type IV [31,32]. Specific surface area of the samples amounted to 19.3 m^2^/g and 110.3 m^2^/g for nHAp and µHAp, respectively. BET adsorption measurements showed the total volume of the pores with their diameter remaining between 0.85 and 70 nm amounts to 0.07 cm^3^/g for nHAp and to 0.56 cm^3^/g for µHAp. In general, the above data showed the superiority of the µHap filler in terms of a surface development. Not only was its specific surface area over five times larger, but also its total pore volume exceeded that of nHAp by a factor of seven.

**Figure 5:**
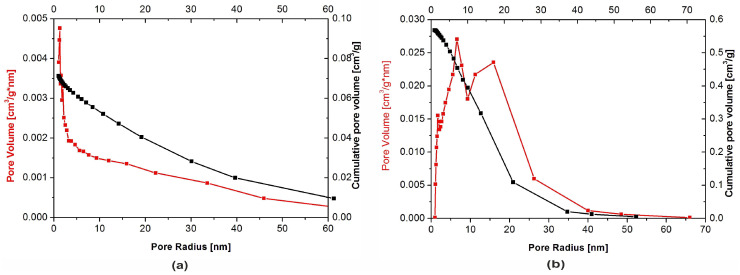
Pore size distribution for: (**a**) Nanosize particles and (**b**) microsize particles powders.

The physical adsorption of gases on microporous solids took place through the formation of a multilayer structure (layer by layer). In the case of mesoporous materials, it followed a volume filling mechanism. For both types of materials, micropore filling occurred at comparatively low values of relative pressure. The formation of an adsorption film on mesoporous surface in that case was followed by the volume filling of the mesopores with a liquid adsorbate. This process is known as a capillary condensation and it occurs at high values of a relative pressure. Dependencies of a pore diameter, determined with the BJH method, on its volume (including the cumulative pore volume) are presented in Figure 5 below.

Although the presence of micro-, meso- and macropores was recorded in both types of the filler, their distribution was significantly different. In the nHAp sample, macropores with a diameter higher than 50 nm occupied a volume of 9.31 × 10^−5^ cm^3^/g·nm. A cumulative volume of mesopores (2–50 nm) amounted to 0.024 cm^3^/g·nm, whereas for nanopores, with a diameter lower than 2 nm size, the volume was equal to 0.026 cm^3^/g·nm. It is also evident from Figure 5a that, among nanopores, those with a diameter equal to 1.1–1.3 nm were predominant.

Macropores with a diameter higher than 50 nm were also present in the µHAp powder. However, their cumulative volume amounted to 1.3 × 10^−4^ cm^3^/g·nm, while mesopores (2–50 nm) occupied a volume of 0.23 cm^3^/g·nm and nanopores that of 0.066 cm^3^/g·nm. As seen in Figure 5b, the dominant mesopores had a diameter of ca. 8 and 18 nm. It can, therefore, be concluded that the volume of mesopores was more than 10-fold greater as compared to that typical for nHAp. The sample also contained pores with a diameter lower than 10 nm but their contribution to the overall porosity was relatively small. From the above results, it appeared that these particles were exceedingly porous, thus having a well-developed surface, furnished with inequalities, depressions, and deep pores.

Methyl methacrylate was used as a monomer for the preparation of polymer composite samples. The molecular size of this compound is about 0.97 nm and, thus, it falls within the range of mesopores. However, the pore volume, considering the monomer adsorption capacity, in nHAp powder is negligible.

### 3.2. Characterization of HAp/PMMA Composites

It is common knowledge that a supplement of HAp, as well as other inorganic materials including such contrast media as ZrO_2_ or BaSO_4_, may lead to both a drop of cross-linking temperature and an increase of setting time of acrylate-based bone cements. Additionally, these parameters are also affected by the size and molecular weight of the acrylate polymer being another component of the bone cement powder. In order to directly assess the effect of HAp on these parameters, the addition of the contrast media was purposefully omitted. In the present work, all the samples were prepared on the basis of the same acrylate copolymer, namely PMMA/PMA, with the variables being a type and concentration of the HAp additive. In both cases, namely those of micro- and nanoscale powders, the HAp concentration varied between 3 and 21 weight percent.

The results of a maximum cross-linking temperature and setting time measurements, including the time to reach the maximum hardness, are presented in Table 2 below. Combining the liquid and powder components of bone cement resulted in the formation of free radicals, which break the double C=C bond in methyl metacrylate and initiated its addition free-radical polymerization reaction leading to the material cross-linking. This reaction was strongly exothermic—under present experimental conditions and for a pure acrylate powder, its temperature amounted to 87.4 °C. The addition of the nanoscale HAp, independent of its concentration, did not cause any substantial changes of that temperature. Dissimilar was the situation for the microHAp additive, where the significant effect of the filler concentration on the reaction temperature was observed. For a composite containing 6 weight % of that additive, this temperature was approximately the same as that for the pure acrylate. The increase of the μHAp concentration to 12 weight % and then to 21 weight % resulted in a drop of the reaction temperature by the component of 20 °C and 42 °C, respectively. The effect of lowering that temperature was beneficial since high temperatures may have resulted in a local tissue necrosis, thus leading to the destruction of a neural ending. From the point of view of safe temperature levels, the most valuable bone cement samples seemed to be those of the highest μHAp concentrations. The results observed indicated that lowering reaction temperature cannot be simply associated with the filler playing the role of a heat accumulator, since it was shown that this temperature was affected by the size of its particles. The larger they are and the higher are their specific surface areas, the more likely it is that they will influence on the cross-linking temperature. This effect may be associated with the lower availability of monomer molecules trapped in μHAp mesopores. Lower availability did not mean different reaction efficiency—the reaction simply needed more time to be completed. Comparable mechanical properties of the samples (see Figure 11) proved the correctness of the above assumption. The differences observed resulted rather from varied filler concentrations than from different mechanism of polymerization. A similar outcome was observed in the case of measurements of time required to attain the maximum reaction temperature. Up to a certain level of concentrations, amounting to approximately 12 weight %, the addition of nHAp did not bring about any prolongation of the time needed to complete the polymer exothermic cross-linking reaction. Only at the concentration of 18 weight %, that time was prolonged by one minute with a similar extension being observed for the sample containing 21 weight % of the filler. Simultaneously performed hardness tests showed that the time required to attain its maximum value was independent of the nHAp concentration and it amounted to ca. 11 min. As opposed to that, the addition of microscale hydroxyapatite filler substantially affected the temperature of an exothermic cross-linking reaction and the time of its attainment. A drop of the maximum reaction temperature by 50 °C and nearly a two-fold extension of the time to reach that maximum were observed as a result of changing the µHAp concentration from 3 to 21 weight %. As far as the time necessary to attain the maximum hardness values was concerned, that for the lowest µHAp concentrations of 3 and 6 weight % did not differ much from the values recorded for the nHAp filler. However, in the case of the higher µHAp content, it began to extend to finally reach the value of 27 min and 29 s for the sample containing 18 weight % of µHAp. This meant that the use of the microscale hydroxyapatite filler substantially reduced the availability of monomer molecules, thus impeding the cross-linking reaction.

**Figure 6:**
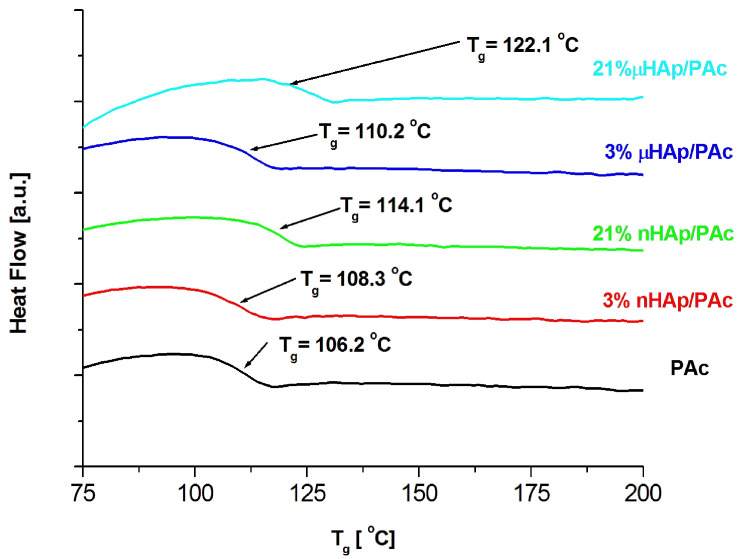
DSC thermograms of the pure polymer matrix and those of the composite materials containing hydroxyapatite filler at different particle size and concentrations.

**Figure 7:**
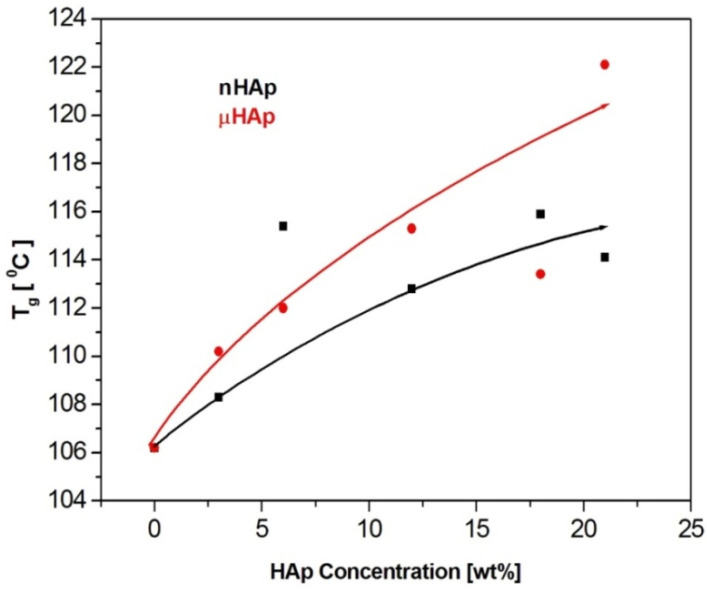
The effect of the filler particle size and concentration on the glass transition temperature of the composite materials.

Examples of DSC thermograms for a nonmodified bone cement as well as those modified with the nano- and microscale hydroxyapatite filler at the concentrations of 3 and 21 weight % are shown in Figure 6 below, while Figure 7 presents the values of glass transition temperature of all investigated samples.

The results presented above show a substantial influence of the hydroxyapatite filler on the glass transition temperature of the obtained composites. It is worth noting that the same amount of the additive may affect the T_g_ value differently, depending on the HAp particle size. At the highest filler concentration, the nanoscale filler increased T_g_ by the magnitude of 6 °C, while the microHAp additive made it grow by 16 grades. A similar behavior was observed in the case of PMMA filled with silicon dioxide [33]. The observed tendency pointed to a strong interaction between monomer molecules and filler particles at the stage of sample preparation. Very likely, this interaction had a polar character. Strong polar interactions between the filler particle and the macromolecule of the polymer severely limited the mobility (elasticity) of the latter, thus increasing its glass transition temperature. As revealed by the BET adsorption data, specific surface areas of the fillers applied substantially differed from one another. The same measurements showed differences in a pore number and size.

The BET results remained in a good agreement with the DSC data. The lower specific surface area of the nHAp filler, as well as its lower pore number and size, affected the filler-monomer interactions to a lesser extent. In contrast to that, the interactions with the monomer of the microsize filler were much stronger. As it was shown by the FTIR analysis, microsize hydroxyapatite contained considerably more polar groups than nHAp. In addition, these groups were less accessible in the case of nHAp since it had a large content of the A fraction of carbonate anions in its structure, substantially lowering the accessibility of hydroxyl moieties. This is a reason why the presence of the microsize filler at the highest concentration of 21 weight % shifted the glass transition temperature of the composite as far up as by 16 degrees.

**Figure 8:**
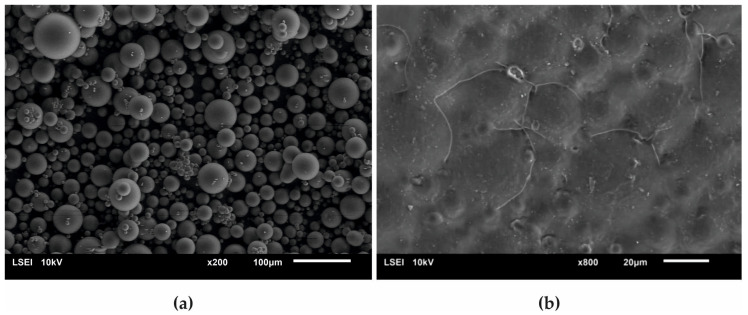
Surface SEM micrographs of (**a**) the initial PAc copolymer and of (**b**) the ready composite cross-linked without any addition of the HAp filler.

Figure 8 presents a SEM micrograph of an initial acrylate copolymer powder (a) and that of polymerized cross-linked bone cement without any addition of hydroxyapatite (b). The size distribution of particles shown in the Figure 8a varied between 2.5 µm and 65.7 µm with the average diameter amounting to 17.5 µm. Following the cross-linking process, one obtained a rather smooth surface with the densely packed, small globules originating from the initial copolymer.

**Figure 9:**
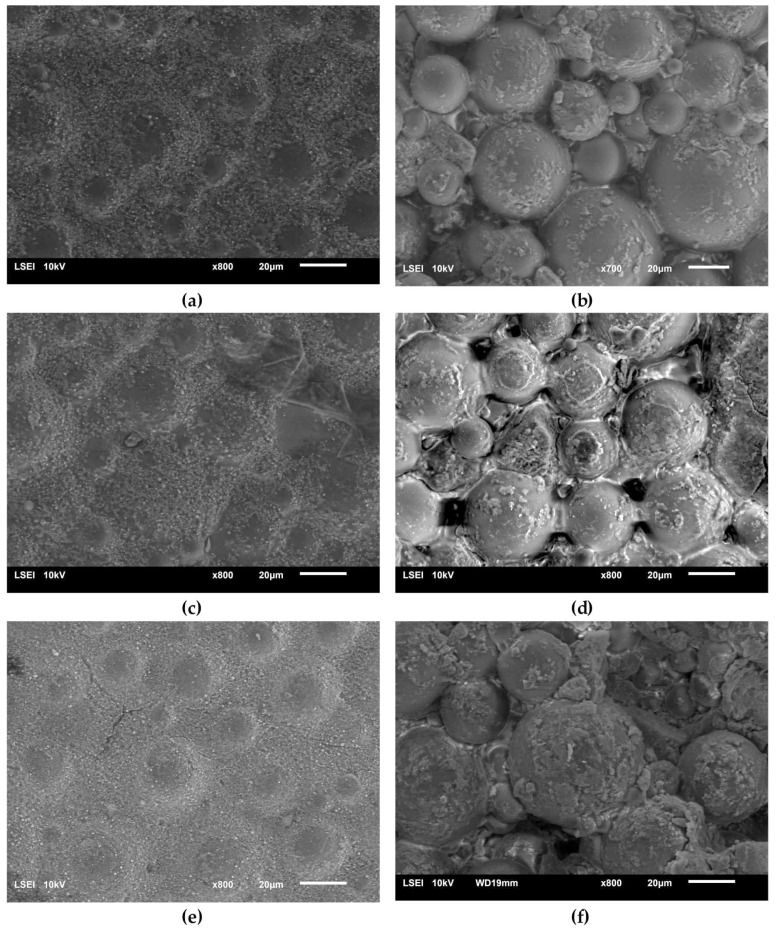
Surface SEM micrographs of the bone cement samples: PAc, 6% nHAp/PAc (**a**), 6% μHAp/PAc (**b**), 12% nHAp/PAc (**c**), 12% μHAp/PAc (**d**), 21% nHAp/PAc (**e**), and 21% μHAp/PAc (**f**).

Figure 9 presents the surface micrographs of composites produced with an addition of the hydroxyapatite filler, either nHAp or μHAp. The surface of nHAp-filled composites was similar to that of the nonmodified copolymer. In the material containing 21 weight % of nHAp, a decreased degree of packing of matrix globules was observed with flat areas emerging between the hills. The addition of μHAp brought about larger modifications of a surface morphology, even at low filler concentrations. Clear boundaries between separated globules began to appear, with their size (remaining in the 5–30 μm range) and packing degree independent of the amount of admixed HAp. It is evident from the above micrographs that the form of the HAp filler had a significant influence on the morphology of the acrylate polymer-based composite materials, which was not only related to the size of the filler grains, but also depended on their specific surface area and the size of the surface pores. The surface of µHAp-enriched composite looked like that of the unmodified copolymer. This was the consequence of the fact that the µHAp filler was characterized by more than twice the specific surface area with a large number of mesopores. Being trapped in these macropores, a substantial fraction of monomer molecules was not available for the cross-linking reactions with the resulting formation of cross-linking chains of lower degree of polymerization. In the course of the reaction, however, the growing macromolecules left the filler pores and began to react with the monomer. This process resulted in the formation of a shell around each globule, whose volume increased with the increasing amount of admixed µHAp. A presence of such elements as (hydroxyapatite originating) calcium and phosphorus in the shell material was confirmed with the help of the EDS analysis.

An opposite situation was encountered in the case of nHAp filler, whose structure and low specific surface area determined the unobstructed availability of the monomer, thus making the cross-linking reaction follow the same mechanism as took place in a nonmodified acrylate bone cement.

**Figure 10:**
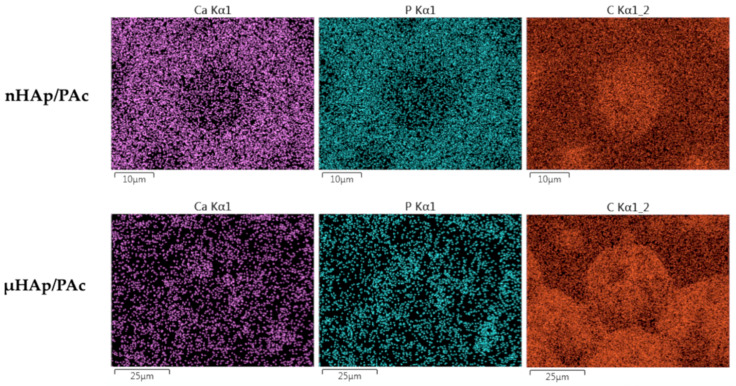
Examples of calcium, phosphorus, and carbon distribution maps in the nHAp/PAc and μHAp/Pac composite materials.

In order to assess the degree of dispersion of the hydroxyapatite filler in a polymer matrix, the surface EDS mapping of the particular composite elements (carbon, calcium, and phosphorus) was performed. Figure 10 presents examples of that mapping for the nHAp/PAc and μHAp/Pac composites with the filler concentration amounting to 21 weight %.

From the above micrographs it appears that the nHAp powder was mainly localized on the globule boundaries. Due to the small diameters of their particles and low specific surface area, in the cross-linking process the powder was pushed down to the newly formed flat part of the polymer matrix. A situation slightly differed for the μHAp/Pac composite, where the degree of a powder dispersion was high. In that case, the filler could be found both on the surface of the globules and on the deeper polymer fractions. This was the result of the powder surface development leading to better distribution between original, globular fragments of the polymer matrix and those formed in the cross-linking process. It should be stressed here that, at the above mentioned sites, both nano- and microparticles showed no tendency to form large aggregates. This was a likely result of the solvatation of nHAp nanoparticles with the molecules of such a polar monomer as methyl methacrylate. Interactions between polar C=O bonds of the monomer and the phosphate groups in nHAp contributed to the better dispersion of the solid particles. In addition, the growing polymer macromolecules surrounded nHAp particles, thus preventing the interaction between these particles. Both effects contributed to the constraint of the aggregation process. The degree of dispersion was slightly higher in the case of composites filled with nHAp, which was a straightforward consequence of the particle size.

**Figure 11:**
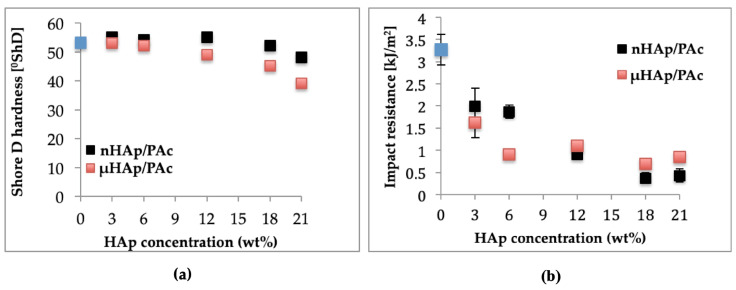
Dependencies of (**a**) hardness and (**b**) impact strength of the composite on the type and concentration of the HAp filler.

Among utility characteristics of bone cements, the most essential are such mechanical properties as hardness and impact resistance. The magnitudes of these parameters as a function of the HAp additive type and its content in acrylate composites are shown in Figure 11, below. Figure 11a presents the respective results of the Shore D hardness (ShD) test. Hardness of the nonmodified acrylate copolymer cement amounted to 53.2 °ShD. The addition of nHAp at 3, 6, and 12 weight % resulted in its slight increase of about 1 °ShD. A rise of the nHAp content to 18 weight % made this parameter decrease to value of 52 °ShD, while its increase by another 3 weight % resulted in further Shore D hardness dropping down to 48 °ShD. All these minor differences were prompted by changes of both surface topography and cross-linking time. To summarize, in the case of the nHAp filler the hardness changes were insignificant from the point of view of the utility properties of acrylate bone cements. Quite different relationships were observed for composites supplemented with microscale HAp. In a way similar to the nanoscale filler, an addition of µHAp at 3, 6, and 12 weight % resulted in hardness figures close to that of a nonmodified cement. Simultaneously, the time of cross-linking reaction was comparable to that of a nonmodified cement. The largest change of hardness (by approximately 15 °ShD) was recorded for the composite containing 21 weight % of the µHAp filler, for which the cross-linking time was nearly twice as long as in the case of the pure acrylate cement. Very likely, these differences were due to a lower degree of polymerization of the cross-linkages resulting from monomer molecules’ adsorption in mesopores and, thus, reducing their availability.

Outcome of the dynamic impact test of manufactured composite materials is shown in Figure 11b. Impact resistance for a nonmodified bone cement amounted to 3.27 kJ/m^2^. Following the addition of 3 and 6 weight % of nHAp, it dropped down to approximately 2 kJ/m^2^ and at 12 weight % it amounted to 0.9 kJ/m^2^, whereas for the highest concentration (21 weight %) it decreased to the magnitude of 0.4 kJ/m^2^. In the case of composites filled with microscale HAp, a larger drop of impact strength was observed at lower filler concentrations attained. Above 12 weight % of the filler, the respective values remained in the range of 1.1 to 0.7 kJ/m^2^.

As seen above, the introduction of filler particles to polymer matrix substantially lowered impact strength of the resulting composites. This effect was connected with insufficient adhesion at the phase boundaries and the size of the interphase area. As was shown earlier in this work, specific surface area of microscale hydroxyapatite was more than five times larger than that of nHAp. This difference resulted in lowering the rate of cross-linking reaction and following the deterioration of the composite mechanical properties. A lower reaction rate signified a decrease of both hardness and impact strength.

## 4. Conclusions

In the present work two types of HAp powders, used as fillers in the manufacture of acrylate bone cements, have been characterized. One of them was a commercial material, in this paper labeled nHAp, of a size span between 0.02 and 2.33 µm, basically comprised of hexagonal HAp with a small addition of TCP and amorphous phases. An addition of this filler, in the concentration range of 3 to 21 weight %, to acrylate copolymer did not affect the magnitudes of cross-linking temperature and time or composite surface morphology. Moreover, it changed its glass transition temperature by only 6 °C. The resulting material exhibited satisfactory mechanical properties in the entire range of the filler concentrations applied.

The second filler was comprised of a self-produced powder, of a size distribution between 2.5 and 85 µm, consisting of hexagonal HAp with a small addition of amorphous phase. Contact angle measurements of the µHAp powder revealed better availability of hydroxyl groups and, therefore, higher water wettability with similar magnitude of surface free energy as in the case of nanosize powder. In addition, the µHAp filler was characterized with five times higher specific surface area and seven times higher porosity than those of nHAp. All these features made the microsize HAp-filled acrylate bone cement substantially different from the nHAp/Pac cements. Increasing the µHAp concentration brought about significant decrease in cross-linking temperature, which is advantageous with regard to the contact with human cells and the prolongation of the composite setting time. Large specific surface area of the filler and its high content of mesopores made the µHAp/Pac composites possess a globular structure and exhibit a drop of their hardness.

Such substantial changes in the properties of both bone cement types, namely those comprised of nHAp/PAc and µHAp/PAc composites, forced a need to assess their interaction with live cells (among other bone cells) and to check how the addition of either nanoscale or microscale filler affects the amount of detrimental MMA monomer released from the cement in the environment simulating pH of the human body fluids. The results of that study will be presented in the next paper published by our research group.

## Figures and Tables

**Table 1:** The values of surface free energy, as well as its polar and dispersion components calculated for nano- and microHAp powders.

Sample	Surface Free Energy [mN/m]	Dispersive Component [mN/m]	Polar Component [mN/m]
nHAp	23.2	20.2	2.9
µHAp	23.5	20.3	3.2

**Table 2:** Results of the cross-linking reaction temperature and time measurements as well as those of the time needed to attain maximum hardness of the composite.

Sample	Maximum Cross-Linking Temperature [°C]	Time to Reach Maximum Temperature	Time to Reach Maximum Hardness
PAc	87.42	06 min 40 s	11 min 30 s
3% nHAp/PAc	90.30	06 min 15 s	11 min 40 s
6% nHAp/PAc	88.98	06 min 28 s	11 min 26 s
12% nHAp/PAc	90.01	06 min 10 s	11 min 50 s
18% nHAp/PAc	88.25	07 min 25 s	11 min 40 s
21% nHAp/PAc	88.98	08 min 35 s	11 min 22 s
3% µHAp/PAc	90.41	06 min 50 s	11 min 45 s
6% µHAp/PAc	93.47	06 min 30 s	11 min 30 s
12% µHAp/PAc	74.12	08 min 02 s	18 min 14 s
18% µHAp/PAc	55.24	10 min 05 s	22 min 20 s
21% µHAp/PAc	41.83	11 min 43 s	27 min 29 s
